# Human adipose tissue–derived mesenchymal stromal cells and their phagocytic capacity

**DOI:** 10.1111/jcmm.17070

**Published:** 2021-12-02

**Authors:** Víctor J. Costela Ruiz, Lucía Melguizo Rodríguez, Rebeca Illescas Montes, Enrique García Recio, Salvador Arias Santiago, Concepción Ruiz, Elvira De Luna Bertos

**Affiliations:** ^1^ Biomedical Group (BIO277) Department of Nursing Faculty of Health Sciences University of Granada Granada Spain; ^2^ Biosanitary Research Institute, ibs Granada Granada Spain; ^3^ Surgical Medical Dermatology and Venereology Service Department of Medicine Virgen de las Nieves Hospital Granada Spain; ^4^ Institute of Neuroscience Centre for Medical Research (CIBM) Health Technology Park (PTS) University of Granada Granada Spain

**Keywords:** immunogenicity, infection, phagocytosis, stem cell‐microenvironment interactions, stromal cells

## Abstract

Mesenchymal stromal cells (MSCs) have evidenced considerable therapeutic potential in numerous clinical fields, especially in tissue regeneration. The immunological characteristics of this cell population include the expression of Toll‐like receptors and mannose receptors, among others. The study objective was to determine whether MSCs have phagocytic capacity against different target particles. We isolated and characterized three human adipose tissue MSC (HAT‐MSC) lines from three patients and analysed their phagocytic capacity by flow cytometry, using fluorescent latex beads, and by transmission electron microscopy, using *Escherichia coli, Staphylococcus aureus* and *Candida albicans* as biological materials and latex beads as non‐biological material. The results demonstrate that HAT‐MSCs can phagocyte particles of different nature and size. The percentage of phagocytic cells ranged between 33.8% and 56.2% (mean of 44.37% ± 11.253) according to the cell line, and a high phagocytic index was observed. The high phagocytic capacity observed in MSCs, which have known regenerative potential, may offer an advance in the approach to certain local and systemic infections.

## Graphical abstract

Study of the phagocytic capacity of human adipose tissue‐derived mesenchymal stromal cells.

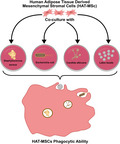

## 1 INTRODUCTION

Mesenchymal stromal cells (MSCs) were first described by Friedenstein et al. as bone marrow cells with fibroblastic morphology and osteogenic character.^1^, ^2^ This non‐hematopoietic undifferentiated cell population derives from the mesoderm with clonogenic character and has proved able to adhere to plastic in *in vitro* cultures. The main characteristics of MSCs are their colony‐forming capacity, adherence to plastic, expansion in *in vitro* cultures, and their pluripotential character, conferring them with potential osteogenic, adipogenic and chondrogenic capacities. They are also characterized by certain surface markers, showing positive CD44, CD73, CD90 and CD105 expression and negative CD14, CD11b, CD19, CD79a, CD34, CD45 and HLA‐DR expression.^3^, ^4^, ^5^, ^6^, ^7^, ^8^

MSCs have been isolated from peripheral blood, umbilical cord, amniotic fluid, placenta, and articular cartilage, among other tissues.^9^, ^10^, ^11^, ^12^, ^13^, ^14^, ^15^ However, hematopoietic and adipose tissues are the most widely used sources due to their easier availability and processing.^5^, ^10^

MSCs have multiple clinical applications, mainly in regenerative medicine, through their capacity to migrate to the focus of damaged tissue and induce its repair or replacement; this capacity derives from their pluripotentiality and their secretion of bioactive substances that can act at both local and systemic level.^16^, ^17^, ^18^ MSCs have also been attributed with potent immunomodulatory properties that may be useful to control inflammation and tissue damage.^19^ They express certain Toll‐like receptors (TLRs), mannose receptors (MRs) and scavenger receptors (SRs), indicating a possible role against infection, although this has yet to be confirmed.^20^, ^21^, ^22^, ^23^ Also, MSCs seem to have the capacity to induce the phagocytic activity of other cells, such as polymorphonuclear cells or alveolar macrophages by releasing.^24^, ^25^ Some other authors have even suggested the use of MSCs in sepsis, through its action at different levels, such as its ability to locate to damage tissue, secrete paracrine signals to systemic and local inflammation, decrease apoptosis, promote neoangiogenesis, stimulate immune cells and show antimicrobial activity.^26^ Later, Khan et al. reported that MSCs are new phagocytic cells with a high potential for immunotherapy in the treatment of tuberculosis.^23^

The isolation, culture, identification, differentiation, function and regenerative capacity of MSCs have been widely studied, prompting considerable interest in their potential therapeutic usefulness in different diseases. In fact, they are among the most frequently used cell types in regenerative medicine, with numerous clinical applications.^23^, ^27^, ^28^, ^29^

Human adipose tissue MSCs (HAT—MSCs) are found in the stromal vascular fraction of subcutaneous adipose tissue. When the cells of this vascular fraction are cultivated in the required culture medium, both adipose stem cells and stromal vascular fraction cells can be obtained.^30^, ^31^ HAT‐MSCs have been used for years in regenerative plastic surgery.^31^, ^32^ In the same way, these cells have also been used for the treatment of anal fistulas, diabetic foot, alopecia and certain defects in soft tissues such as healing processes, among others.^31^, ^33^, ^34^, ^35^, ^36^ Thus, it has also been possible to demonstrate the regenerative capacity of HAT‐MSCs in combination with platelet‐rich plasma in soft tissue regeneration and wound healing.^32^, ^37^ On the contrary, the anti‐inflammatory and immunomodulatory potential of this cell population has been pointed out as a possible cell therapy in infection caused by the SARS‐CoV‐2 virus.^38^

With this background, the objective of this study was to determine the phagocytic capacity of MSCs by means of two study techniques (flow cytometry and transmission electron microscopy), using target particles of different size and origin, such as latex beads and microorganisms. This will contribute to the knowledge of the physiology of this cell population.

## 2 MATERIAL AND METHODS

### 2.1 Ethical procedures

This study was approved by the Ethical Committee for Biomedical Research of Granada, Spain (CEIM/CEI Granada; Reg. code 1491‐N‐18) and by the Coordinating Committee of Ethical Biomedical Research of the Andalusian Autonomous Community (CCEIBA; Reg. code VJCR 16/41141). This approval was granted for the collection and processing of tissue samples. Procedures were performed in accordance with the 1964 Helsinki declaration (Ethical Principles for Medical Research Involving Human Subjects) and its 7 later amendments. Informed consent was signed by all donors.

### 2.2 Establishment of human adipose tissue MSCs

HAT‐MSC lines were isolated from three adipose tissue samples from two females aged 40 and 47 years and one male aged 52 years. Cells were provided by the Tissue Bank (Biobank) of the Andalusian Public Health System. All three patients signed their informed consent to the study of their samples. Samples were digested as follows: after initial washing with phosphate‐buffered saline (PBS), they were ground and then treated with 0.15% collagenase type I (Sigma, USA) for 60 min at 37°C under agitation with a double mixer. Collagenase was subsequently inactivated by using basal culture medium (Dulbecco's Modified Eagle's Medium supplemented with 10% foetal bovine serum [FBS]: DMEM + 10% FBS) and centrifuging for 10 min at 309 *g*. The sediment was suspended in DMEM + 10% FBS, and the cell suspension obtained was cultured in 75‐cm^2^ culture flasks at a density of 2 × 10^3^ cells/cm^2^ at 37°C in 5% CO_2_ atmosphere under standard conditions.^39^, ^40^

Adherent cells were washed at 48 h with fresh medium and cultured at 37°C and 5% CO_2_, changing the culture medium three times a week. Passage 1 was performed once 85%–90% cell confluence was obtained (7–10 days), typifying the cell line by flow cytometry to ensure the presence of MSC‐characteristic surface markers (monoclonal antibody from BD Pharmigen TM, Madrid, Spain) with positivity for CD44, CD73, CD90 and CD105 and negativity for CD11b, CD19, CD45 and HLA‐DR (Table S1). At the same passage, cells were differentiated to osteogenic and adipogenic lineage, following the protocol of Zajdel et al.^41^ and Munir et al.^42^ respectively.

For the experiments, cells from each donor were used at passages 3–4.

### 2.3 Study of phagocytic capacity

The phagocytic capacity of HAT‐MSCs was analysed by flow cytometry and transmission electron microscopy.

#### 2.3.1 Flow cytometry

Phagocytic activity was studied by flow cytometry. Cultures of HAT‐MSC were detached from the cultured flask at passage 3 or 4 using a solution of 0.05% (w/v) trypsin and 0.02% (w/v) EDTA and were then washed and suspended in complete culture medium with 20% FBS at 1 × 10^6^ cells/ml. One hundred microliters of cell suspension was incubated with 2 μl carboxylated FICT‐labelled latex beads (Sigma Aldrich, St Louis, USA) of 2 μm diameter, for 30 min at 37°C in darkness. Cells were washed, suspended in 1 ml PBS and immediately analysed in flow cytometer (FASC Canto II [software Diva 6 V3.1], Becton Dickinson Palo Alto, California, USA). Control assays were carried out at 4°C. The percentage of fluorescence cells was calculated from counts of 2000 to 3000 cells. Results were obtained as the percentage of cells positive for phagocytosis and the mean channel fluorescence, which correlates with the number of particles phagocytosed.^43^

#### 2.3.2 Transmission electron microscopy

HAT‐MSC phagocytic activity was also analysed by transmission electron microscopy. At passages 3–4, cells were placed in 6‐well plates at a concentration of 2 × 10^4^ cells/ml and cultured in medium with 20% FBS at 37°C in 5% CO_2_ atmosphere. The culture medium was removed at 24 h, adding 5 ml fresh medium to each well. Next, 10 µl of DMEM + 10% FBS suspension containing target particles was added to the test wells and 10 µl of DMEM + 10% FBS alone to the control wells. Suspensions of the target particles (latex, *Escherichia coli, Staphylococcus aureus* and *Candida albicans*) were prepared in complete culture medium, always adjusted to 0.5 of the McFarland turbidity standard scale^43^ and then cultured at 37°C in 5% CO_2_ atmosphere. Cells were then cultured and treated following the protocol described by Ruiz et al.^44^

### 2.4 Statistical analysis

R software (version 2.9.2, Auckland, New Zealand) was used for data analyses of phagocytosis by flow cytometry. Phagocytic capacity was compared using the Student's *t* test. *p* ≤ 0.05 was considered statistically significant in all tests. At least three experiments were performed in all assays and for each culture. Data were expressed as means ± standard deviation (SD).

## 3 RESULTS

**FIGURE 1:**
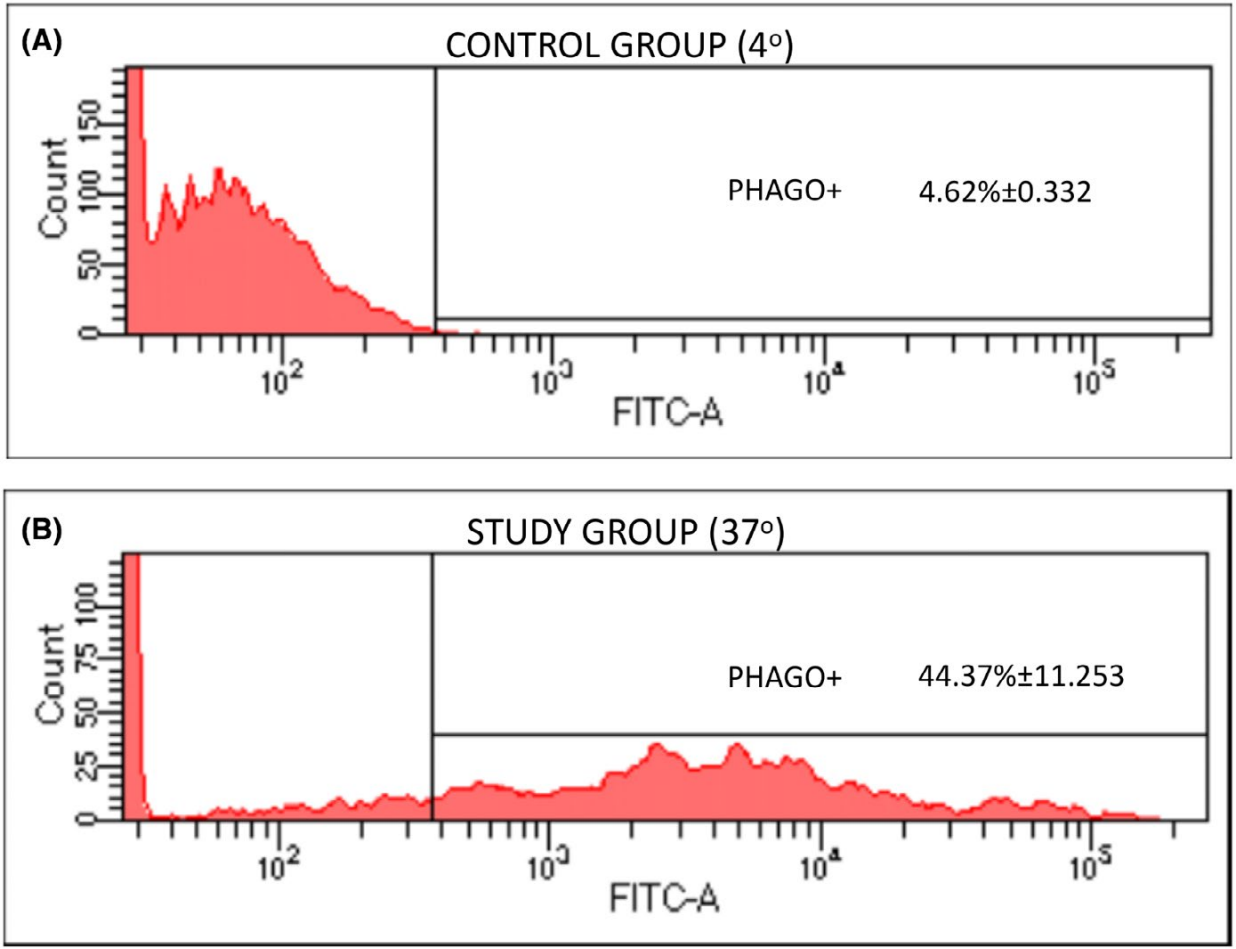
Fluorescence histogram obtained by analysing the phagocytic capacity of HAT‐MSCs using the flow cytometry technique. (A) Incubated with FITC‐labelled latex beads at 4°C (control). (B) Incubated with FITC‐labelled latex beads at 37°C (study group). This figure shows the results of one of the experiments

Flow cytometry showed that a high percentage of the HAT cells isolated and characterized as MSCs had phagocytic capacity ranging from 33.8% to 56.2% (44.37% ± 11.253), according to the cell line. The mean channel fluorescence was also elevated in all three lines, ranging between 4314 and 4973 (4619 ± 0.332), indicating a high phagocytic index (Figure 1; Table 1).

**TABLE 1:** Phagocytic capacity of HAT‐MSCs determined by flow cytometry analysis

Samples/Phagocytosis conditions	Expression			Fluorescence intensity		
	%	SD	*p*	Mean	SD	*p*
Control group (4°C)	4.62	0.332	—	1.313	0.134	—
Study group (37°C)	44.37	11.253	0.004*	4.619	0.332	0.001*

Note

Results are expressed as mean ± SD and percentage of cells that have phagocytic capacity as well as the mean ± SD of the average value of fluorescence intensity. Comparisons of data between study group and control group were evaluated by Student's *t* test. At least three experiments were performed in all assays and for each culture.

Abbreviation: SD, standard deviation.

* *p* ≤ 0.05 significant difference.

**FIGURE 2:**
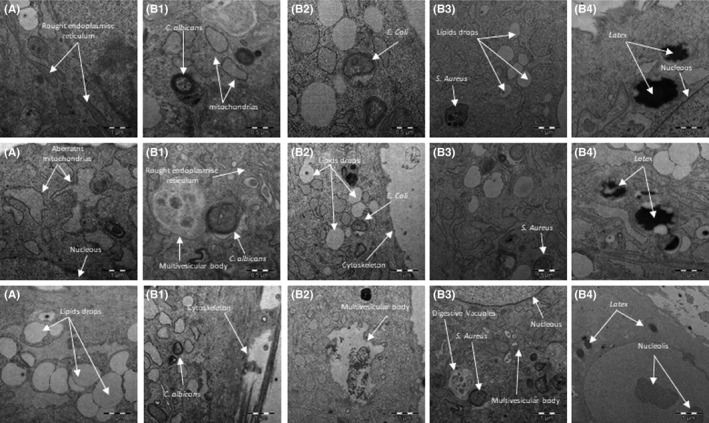
Transmission electron microscopy study of HTA‐MSCs cells cultured with different target particles. (A) HAT‐MSCs in culture (control, without target particles): (B) HAT‐MSC incubated for 24 h at 37°C with: (B1) *C*. *albicans*; (B2) *E*. *coli*; (B3) *S*. *aureus*; or (B4) latex beads. Different cellular elements (cytoskeleton, mitochondria, rough endoplasmic reticulum, nucleus and nucleoli) are also pointed out in figures

**FIGURE 3:**
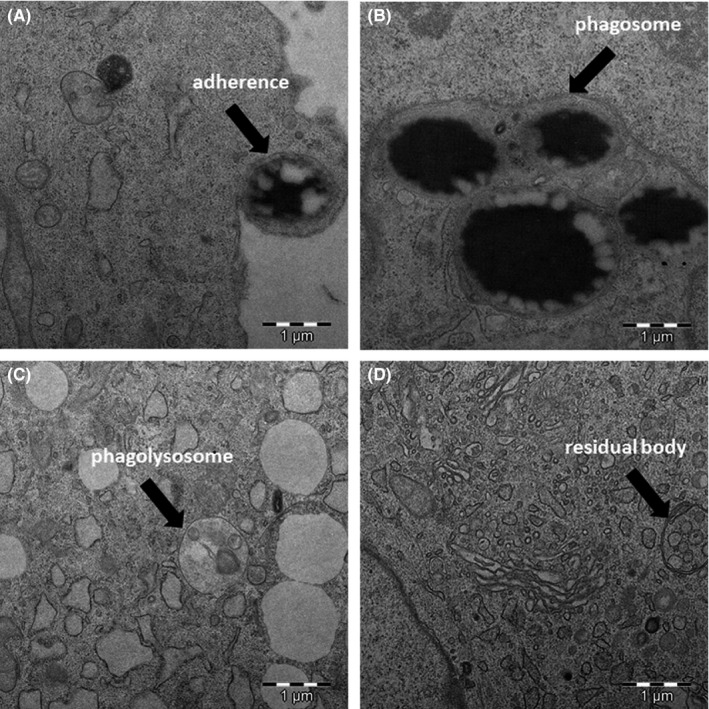
Transmission electron micrographs of HAT‐MSCs incubated for 24 h at 37°C with target particles, showing the different stages of the phagocytic process. (A) Adherence/binding; (B) phagosome; (C) phagolysosome; and (D) residual body

**FIGURE 4:**
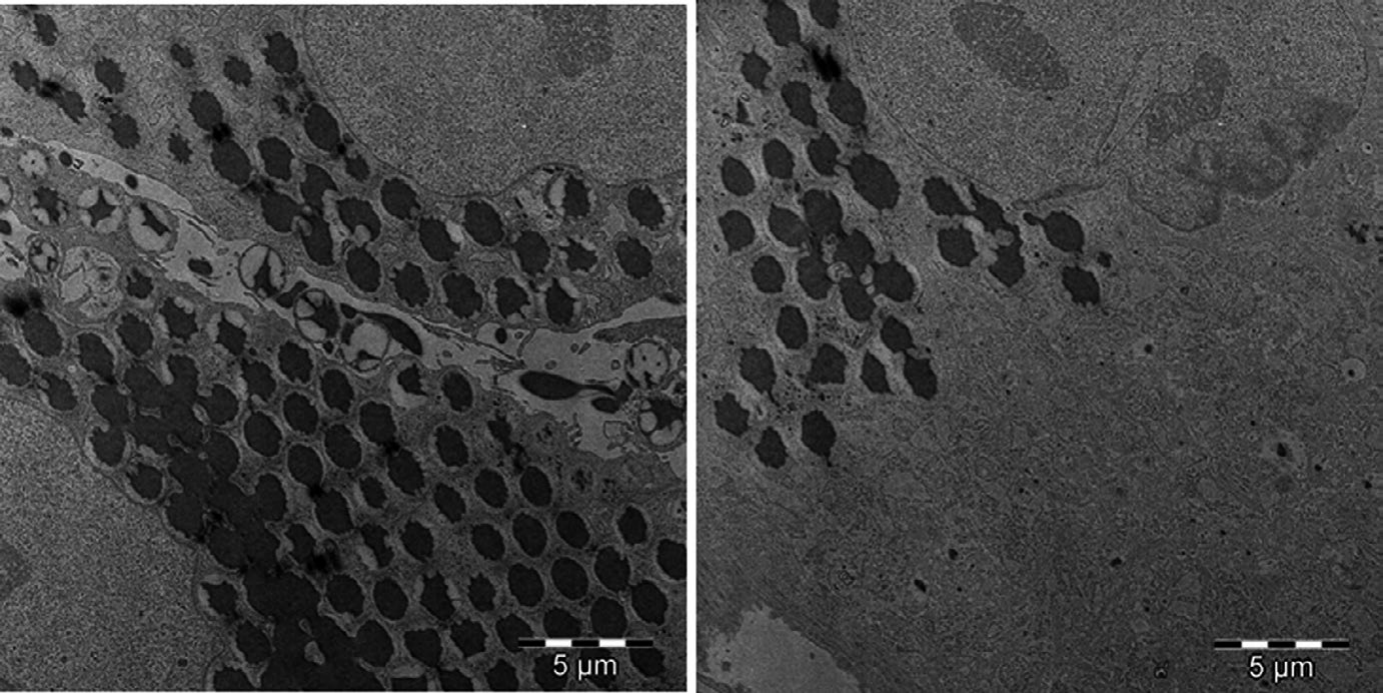
Phagocytosis of latex particles by HAT‐MSCs incubated for 24 h at 37°C and analysed by transmission electron microscopy

Transmission electron microscopy confirmed the results obtained by flow cytometry, demonstrating the capacity of the cells to internalize the target particles under study and ruling out the possibility that the detected fluorescence derived from the adhesion of latex beads to the cell surface. The images in Figure 2 depict the ultrastructure of HAT‐MSCs (pronounced cytoskeleton, abundant mitochondria, rough endoplasmic reticulum, nucleus with two prominent nucleoli, etc.), the ultrastructure of the same cells in culture and after incubation with biological (*C. albicans; E. coli; and S. aureus*) or synthetic (latex beads) particles. It is observed abundant lipid drops, as a consequence of the origin of the line; the presence of multivesicular bodies, residues of the digestion of the biological material (microorganisms); and the phagosomes containing the phagocytosed target particle inside. Figure 3 shows micrographs of HAT‐MSC incubated for 24 h with various target particles, showing different stages of the phagocytic process with adhesion, engulfment, phagosomes and multivesicular bodies. The high phagocytic index observed is noteworthy, especially when cells were cultured with latex beads (Figure 4).

## 4 DISCUSSION

This study demonstrated the phagocytic capacity against different target particles of cells isolated from HAT samples and characterized as MSCs.

Flow cytometry studies confirmed the capacity of a high percentage of HAT‐MSCs to phagocytose latex beads, large numbers of which were ingested by the cells. These data are consistent with reports of the same functional capacity for other cell populations that differentiate from MSCs, including osteoblasts, cells of mesenchymal origin that derive from osteoprogenitors.^44^, ^45^, ^46^ In the osteoblast, this phagocytic activity can be modulated by biological factors, drugs or physical agents.^47^, ^48^, ^49^ Preadipocytes and adipocytes, also of mesenchymal origin, express a wide spectrum of TLRs, and preadipocytes can become macrophage‐like cells.^50^ TLR2 is expressed in early stages of adipocyte differentiation and TLR9 in later stages of this process. In addition, TLR2 synthesis can be stimulated by bacterial lipopolysaccharides (LPS), zymosan from yeast cell wall, and murine or human adipocyte tumour growth factor.^51^

Flow cytometry is a fast and simple technique that yields a large amount of data on a cell population, including functional information, allowing quantification of its phagocytic capacity and the higher or lower number of particles that a cell can phagocytose according to the fluorescence intensity observed. However, non‐specific binding to the cell surface can give rise to false positives, even when the appropriate negative controls are used.^52^ For this reason, the most rigorous method to confirm this functional capacity in a population, ruling out false positives, is to incubate cells in the presence of a target particle and then analyse the cells by transmission electron microscopy. This offers visualization of the internalized particles, the phagosome and the degree of digestion of the particle it contains.

Our transmission electron microscopy results show the ultrastructure of HAT‐MSCs, as previously described Pasquinelli et al.^53^ in MSCs from different origins. Moreover, we verified the capacity of HAT‐MSCs to phagocytose both non‐biological (latex) and biological (microorganisms) materials. Thus, the technique also confirmed the high phagocytic index observed by flow cytometry, especially when the target particles were latex. The biological targets included one Gram‐negative and one Gram‐positive bacterium and one yeast. These microorganisms have been selected for their structural and size differences, as well as for being prevalent microorganisms in infectious processes.

Khan et al. recently reported that MSCs isolated from human bone marrow or umbilical cord can phagocytose *Mycobacterium tuberculosis* (Mtb).^23^ For this purpose, bacteria marked with a green fluorescent protein (gfp) were incubated with MSCs and subsequently analysed by fluorescence microscopy. However, the authors acknowledged that the Mtb uptake mechanisms are unclear. In contrast to other phagocytic cells, there are fewer known receptors in MSCs that could be involved in particle uptake. At least two types of SR (MARCO and SR‐B1) appear to mediate in the uptake of Mtb but not of MRs in MSCs. Thus, a marked inhibition of Mtb uptake was observed in MSCs treated with a combination of antibodies against MARCO and SR‐B1, although a role for other receptors was not ruled out.^23^ In this line, treatment with LPS, which activate TLR‐4, was found to induce osteogenic differentiation in MSCs, while dsRNA activated TLR‐3 and improved stem cell migration.^22^

Other studies show how MSCs can produce factors with bactericidal activity^54^, ^55^; act synergistically with antibiotics to generate bactericidal activity^56^, ^57^; alter the expression of antimicrobial peptides and the production of cytokines, through their activation by innate immune pathways^58^; influence on the formation of the biofilm^55^, ^59^; and stimulate phagocytosis by neutrophils.^55^

Although the action of MSCs against infection is not well documented, they have been attributed with an immunomodulatory role in both the innate and adaptive response through their effects on numerous cell types, including neutrophils, dendritic cells, Natural Killer cells, B lymphocytes, T lymphocytes and regulatory T cells.^60^, ^61^, ^62^

With regard to the immunological role of adipose tissue stem cells, this population has an immunomodulatory character in tumour immune response processes,^63^, ^64^ as well as in certain immunological inflammatory processes in wound healing.^65^, ^66^ Recent studies have revealed the immune‐mediating capacity of both adipose tissue stem cells and vascular fraction stem cells in COVID‐19 disease.^38^, ^67^, ^68^ In this sense, an in vivo study detected how MSCs administered intravenously can promote an increase in peripheral blood lymphocytes, a decrease in C‐reactive protein and a decrease in certain cell groups responsible for the release of pro‐inflammatory cytokines in patients infected by SARS‐CoV‐2.^69^ Thus, and considering the possible phagocytic capacity and the different immunological properties of HAT‐MSCs, its use in certain inflammatory processes, such as infectious processes, may represent an important advance in the approach and treatment of certain pathologies.

Our demonstration of the phagocytic capacity of HAT‐MSCs supports the immunomodulatory role of these cells and indicates the need for more detailed research on this function, its possible clinical relevance and its action on other cell populations.

In summary, HAT‐MSCs have phagocytic capacity against different microorganisms and against non‐biological material. MSCs are known to have major therapeutic potential for regeneration and for the treatment of infections. These new data on their functional capacity expands our knowledge of MSCs and their potential clinical relevance.

## CONFLICT OF INTEREST

The authors declare no competing or financial interests.

## AUTHOR CONTRIBUTIONS

**Victor J. Costela‐Ruiz:** Data curation (equal); Formal analysis (equal); Investigation (equal); Project administration (equal); Resources (equal); Validation (equal); Visualization (equal); Writing – original draft (equal); Writing – review & editing (equal). **Lucía J. Melguizo Rodríguez:** Data curation (equal); Methodology (equal); Validation (equal); Visualization (equal); Writing – review & editing (equal). **Rebeca J. Illescas Montes:** Data curation (equal); Methodology (equal); Validation (equal); Visualization (equal); Writing – review & editing (equal). **Enrique J. García Recio:** Data curation (equal); Investigation (equal); Methodology (equal); Validation (equal); Visualization (equal); Writing – review & editing (equal). **Salvador J. Arias Santiago:** Data curation (equal); Formal analysis (equal); Investigation (equal); Methodology (equal); Validation (equal); Visualization (equal); Writing – review & editing (equal). **Concepción Ruiz:** Conceptualization (equal); Data curation (equal); Formal analysis (equal); Supervision (equal); Validation (equal); Visualization (equal); Writing – original draft (equal); Writing – review & editing (equal). **Elvira J. De Luna Bertos:** Conceptualization (equal); Data curation (equal); Formal analysis (equal); Supervision (equal); Validation (equal); Visualization (equal); Writing – original draft (equal); Writing – review & editing (equal).

## Supporting information

Table S1

## Data Availability

Data available on request from the authors.
