# Supplementary material for: Human adipose tissue–derived mesenchymal stromal cells and their phagocytic capacity
Source: J Cell Mol Med. 2021 Dec 2;26(1):178–85. doi: 10.1111/jcmm.17070 (PMC8742185; doi:10.1111/jcmm.17070)
Supplement: Supplementary file 1 — Table S1 [file JCMM-26-178-s001.doc]

**Table S1**.- Monoclonal antibodies (BD Pharmigen^TM^, Madrid, Spain) used to study antigenic phenotype on cultured HAT-MSC, with their specificity, the fluorochrome used to label the antibody, the supplier, the catalogue number, as well as the citation (below the table). FITC: Fluorescein-isothiocyanate. PE: Phycoerythrin. APC: Allophycocyanin.

| **MAB** | **CD/Specificity** | **Fluorochrome** | **Supplier** | **Catalogue number** | **Citation** |
| --- | --- | --- | --- | --- | --- |
| Control PE | - | PE | BD Pharmingen^TM^ | 559320 | - |
| Control FITC | - | FITC | BD Pharmingen^TM^ | 554647 | - |
| Control APC | - | APC | BD Pharmingen^TM^ | 550882 | - |
| PE Mouse Anti-Human CD11b | CD11b | PE | BD Pharmingen^TM^ | 557321 | See references below |
| FITC Mouse Anti-Human CD19 | CD19 | FITC | BD Pharmingen^TM^ | 555412 | See references below |
| PE Mouse Anti-Human CD44 | CD44 | PE | BD Pharmingen^TM^ | 555479 | See references below |
| FITC Mouse Anti-Human CD45 | CD45 | FITC | BD Pharmingen^TM^ | 555482 | See references below |
| PE Mouse Anti-Human CD73 | CD73 | PE | BD Pharmingen^TM^ | 550257 | See references below |
| APC Mouse Anti-Human CD90 | CD90 | APC | BD Pharmingen^TM^ | 559869 | See references below |
| PE Mouse anti-Human CD105 | CD105 | PE | BD Pharmingen^TM^ | 560839 | See references below |
| FITC Mouse Anti-Human HLA-DR | HLA-DR | FITC | BD Pharmingen^TM^ | 555811 | See references below |

**REFERENCES:**

- **CD11b**:
  - Barclay NA, Brown MH, Birkeland ML, et al, ed. The Leukocyte Antigen FactsBook. San Diego, CA: Academic Press; 1997; .
  - Knapp W. W. Knapp .. et al., ed. Leucocyte typing IV : white cell differentiation antigens. Oxford New York: Oxford University Press; 1989; :1-1182.
- **CD19:**
  - Bradbury LE, Goldmacher VS, Tedder TF. The CD19 signal transduction complex of B lymphocytes. Deletion of the CD19 cytoplasmic domain alters signal transduction but not complex formation with TAPA-1 and Leu 13. J Immunol. 1993; 151(6):2915-2927.
  - Favaloro EJ, Moraitis N, Koutts J, Exner T, Bradstock KF. Endothelial cells and normal circulating haemopoietic cells share a number of surface antigens. Thromb Haemost. 1989; 61(2):217-224.
  - Knapp W. W. Knapp .. et al., ed. Leucocyte typing IV : white cell differentiation antigens. Oxford New York: Oxford University Press; 1989; :1-1182.
  - Nadler LM, Anderson KC, Marti G, et al. B4, a human B lymphocyte-associated antigen expressed on normal, mitogen-activated, and malignant B lymphocytes. J Immunol. 1983; 131(1):244-250.
  - Schlossman SF. Stuart F. Schlossman .. et al., ed. Leucocyte typing V : white cell differentiation antigens : proceedings of the fifth international workshop and conference held in Boston, USA, 3-7 November, 1993. Oxford: Oxford University Press; 1995; .
  - Uckun FM, Muraguchi A, Ledbetter JA, et al. Biphenotypic leukemic lymphocyte precursors in CD2+CD19+ acute lymphoblastic leukemia and their putative normal counterparts in human fetal hematopoietic tissues. Blood. 1989; 73(4):1000-1015.
- **CD44:**
  - Galandrini R, Galluzzo E, Albi N, Grossi CE, Velardi A. Hyaluronate is costimulatory for human T cell effector functions and binds to CD44 on activated T cells. J Immunol. 1994; 153(1):21-31.
  - Günthert U. CD44: a multitude of isoforms with diverse functions. Curr Top Microbiol Immunol. 1993; 184:47-63.
  - Patel DD, Liao HX, Haynes BF. CD44 workshop panel report. In: Kishimoto T. Tadamitsu Kishimoto .. et al., ed. Leucocyte typing VI : white cell differentiation antigens : proceedings of the sixth international workshop and conference held in Kobe, Japan, 10-14 November 1996. New York: Garland Pub.; 1997; :373-375.
  - Schlossman SF. Stuart F. Schlossman .. et al., ed. Leucocyte typing V : white cell differentiation antigens : proceedings of the fifth international workshop and conference held in Boston, USA, 3-7 November, 1993. Oxford: Oxford University Press; 1995; .
  - Stamenkovic I, Amiot M, Pesando JM, Seed B. A lymphocyte molecule implicated in lymph node homing is a member of the cartilage link protein family. Cell. 1989; 56(6):1057-1062.
- **CD45:**
  - Bradstock KF, Janossy G, Pizzolo G, et al. Subpopulations of normal and leukemic human thymocytes: an analysis with the use of monoclonal antibodies. J Natl Cancer Inst. 1980; 65(1):33-42.
  - Hermiston ML, Xu Z, Weiss A. CD45: a critical regulator of signaling thresholds in immune cells. Annu Rev Immunol. 2003; 21:107-137.
  - Knapp W. W. Knapp .. et al., ed. Leucocyte typing IV : white cell differentiation antigens. Oxford New York: Oxford University Press; 1989; :1-1182.
  - Loken MR, Brosnan JM, Bach BA, Ault KA. Establishing optimal lymphocyte gates for immunophenotyping by flow cytometry. Cytometry. 1990; 11(4):453-459.
  - Terry LA, Brown MH, Beverley PC. The monoclonal antibody, UCHL1, recognizes a 180,000 MW component of the human leucocyte-common antigen, CD45. Immunology. 1988; 64(2):331-336.
  - Terstappen LW, Levin J. Bone marrow cell differential counts obtained by multidimensional flow cytometry. Blood Cells. 1992; 18(2):311-330.
  - Zola H. Leukocyte and stromal cell molecules: the CD markers. Hoboken, N.J.: Wiley-Liss; 2007;
- **CD73:**
  - Airas L, Salmi M, Jalkanen S. Lymphocyte-vascular adhesion protein-2 is a novel 70-kDa molecule involved in lymphocyte adhesion to vascular endothelium. J Immunol. 1993; 151(8):4228-4238.
  - Salazar-Gonzalez JF, Moody DJ, Giorgi JV, Martinez-Maza O, Mitsuyasu RT, Fahey JL. Reduced ecto-5'-nucleotidase activity and enhanced OKT10 and HLA-DR expression on CD8 (T suppressor/cytotoxic) lymphocytes in the acquired immune deficiency syndrome: evidence of CD8 cell immaturity. J Immunol. 1985; 135(3):1778-1785.
  - Schlossman SF. Stuart F. Schlossman .. et al., ed. Leucocyte typing V : white cell differentiation antigens : proceedings of the fifth international workshop and conference held in Boston, USA, 3-7 November, 1993. Oxford: Oxford University Press; 1995; .
  - Thomson LF, Ruedi JM, Glass A, et al. Production and characterization of monoclonal antibodies to the glycosyl phosphatidylinositol-anchored lymphocyte differentiation antigen ecto-5'-nucleotidase (CD73). Tissue Antigens. 1990; 35(1):9-19.
- **CD90:**
  - Baum CM, Weissman IL, Tsukamoto AS, Buckle AM, Peault B. Isolation of a candidate human hematopoietic stem-cell population. Proc Natl Acad Sci U S A. 1992; 89(7):2804-2808. [View reference](http://www.ncbi.nlm.nih.gov/pubmed/1372992)
  - Craig W, Kay R, Cutler RL, Lansdorp PM. Expression of Thy-1 on human hematopoietic progenitor cells. J Exp Med. 1993; 177(5):1331-1342. [View reference](http://www.ncbi.nlm.nih.gov/pubmed/7683034)
  - Knapp W. W. Knapp .. et al., ed. Leucocyte typing IV : white cell differentiation antigens. Oxford New York: Oxford University Press; 1989; :1-1182.
  - Lansdorp PM, Thomas TE. AP Gee, ed. Bone Marrow Processing and Purging. Boca Raton FL: CRC Press; 1991; .
  - Schlossman SF. Stuart F. Schlossman .. et al., ed. Leucocyte typing V : white cell differentiation antigens : proceedings of the fifth international workshop and conference held in Boston, USA, 3-7 November, 1993. Oxford: Oxford University Press; 1995; .
- **CD105:**
  - Gougos A, Letarte M. Identification of a human endothelial cell antigen with monoclonal antibody 44G4 produced against a pre-B leukemic cell line. J Immunol. 1988; 141(6):1925-1933.
  - Lastres P, Bellon T, Cabañas C, et al. Regulated expression on human macrophages of endoglin, an Arg-Gly-Asp-containing surface antigen. Eur J Immunol. 1992; 22(2):393-397.
  - Tomchuck SL, Zwezdaryk KJ, Coffelt SB, Waterman RS, Danka ES, Scandurro AB. Toll-like receptors on human mesenchymal stem cells drive their migration and immunomodulating responses. Stem Cells. 2008; 26(1):99-109.
  - Wang JM, Kumar S, Pye D, van Agthoven AJ, Krupinski J, Hunter RD. A monoclonal antibody detects heterogeneity in vascular endothelium of tumours and normal tissues. Int J Cancer. 1993; 54(3):363-370.
  - Westphal JR, Willems HW, Schalkwijk CJ, Ruiter DJ, de Waal RM. A new 180-kDa dermal endothelial cell activation antigen: in vitro and in situ characteristics. J Invest Dermatol. 1993; 100(1):27-34.
- **HLA-DR:**
  - Barclay NA, Brown MH, Birkeland ML, et al, ed. The Leukocyte Antigen FactsBook. San Diego, CA: Academic Press; 1997; .
  - Dieckmann D, Plottner H, Berchtold S, Berger T, Schuler G. Ex vivo isolation and characterization of CD4(+) CD25(+) T cells with regulatory properties from human blood. J Exp Med. 2001; 193(11):1303-1310.
  - Herodin F, Thullier P, Garin D, Drouet M. Nonhuman primates are relevant models for research in hematology, immunology and virology. Eur Cytokine Netw. 2005; 16(2):104-116.
  - Ibisch C, Pradal G, Bach JM, Lieubeau B. Functional canine dendritic cells can be generated in vitro from peripheral blood mononuclear cells and contain a cytoplasmic ultrastructural marker. J Immunol Methods. 2005; 298(1-2):175-82.
  - Kitani A, Chua K, Nakamura K, Strober W. Activated self-MHC-reactive T cells have the cytokine phenotype of Th3/T regulatory cell 1 T cells. J Immunol. 2000; 165(2):691-702.
  - Moran TP, Collier M, McKinnon KP, Davis NL, Johnston RE, Serody JS. A novel viral system for generating antigen-specific T cells. J Immunol. 2008; 175(5):3431-3438.
  - Pawelec G, Ziegler A, Wernet P. Dissection of human allostimulatory determinants with cloned T cells: stimulation inhibition by monoclonal antibodies TU22, 34, 35, 36, 37, 39, 43, and 58 against distinct human MHC class II molecules. Hum Immunol. 1985; 12(3):165-176.
  - Pawelec GP, Shaw S, Ziegler A, Muller C, Wernet P. Differential inhibition of HLA-D- or SB-directed secondary lymphoproliferative responses with monoclonal antibodies detecting human Ia-like determinants. J Immunol. 1982; 129(3):1070-1075.
  - Podolin PL, Bolognese BJ, Carpenter DC, et al. Inhibition of invariant chain processing, antigen-induced proliferative responses, and the development of collagen-induced arthritis and experimental autoimmune encephalomyelitis by a small molecule cysteine protease inhibitor. J Immunol. 2008; 180(12):7989-8003.
  - Sorg RV, Kogler G, Wernet P. Identification of cord blood dendritic cells as an immature CD11c- population. Blood. 1999; 93(7):2302-2307.
  - Ziegler A, Heinig J, Muller C, et al. Analysis by sequential immunoprecipitations of the specificities of the monoclonal antibodies TU22,34,35,36,37,39,43,58 and YD1/63.HLK directed against human HLA class II antigens. Immunobiology. 1986; 171(1-2):77-92.
  - Ziegler A, Uchańska-Ziegler B, Zeuthen J, Wernet P. HLA antigen expression at the single cell level on a K562 X B cell hybrid: an analysis with monoclonal antibodies using bacterial binding assays.. Somatic Cell Genet. 1982; 8(6):775-89.
  - Zola H. Leukocyte and stromal cell molecules : the CD markers. Hoboken, N.J.: Wiley-Liss; 2007.
